# User Adaptive Text Predictor for Mentally Disabled Huntington's Patients

**DOI:** 10.1155/2016/3054258

**Published:** 2016-02-23

**Authors:** Julius Gelšvartas, Rimvydas Simutis, Rytis Maskeliūnas

**Affiliations:** ^1^Automation Department, Faculty of Electrical and Electronics Engineering, Kaunas University of Technology, Studentų g. 50-154, LT-51368 Kaunas, Lithuania; ^2^Department of Multimedia Engineering, Faculty of Informatics, Kaunas University of Technology, Studentų g. 50-414a, LT-51368 Kaunas, Lithuania

## Abstract

This paper describes in detail the design of the specialized text predictor for patients with Huntington's disease. The main aim of the specialized text predictor is to improve the text input rate by limiting the phrases that the user can type in. We show that such specialized predictor can significantly improve text input rate compared to a standard general purpose text predictor. Specialized text predictor, however, makes it more difficult for the user to express his own ideas. We further improved the text predictor by using the sematic database to extract synonym, hypernym, and hyponym terms for the words that are not present in the training data of the specialized text predictor. This data can then be used to compute reasonable predictions for words that are originally not known to the text predictor.

## 1. Introduction

Assistive technologies that enable disabled people to communicate can greatly improve their quality of life. These technologies have to integrate multiple sensors and intelligent software packages. Text predictor is one of the most important components of assistive communication technologies. It has been shown in [1] that text predictor can greatly improve text input rates. People with Huntington's disease usually cannot communicate using spoken or sign language. Assistive technologies are the main tools allowing Huntington's patients to express their ideas. Having efficient text input technologies is therefore highly desirable.

Text predictor is a system that predicts the next block of characters (letters, syllables, words, sentences, etc.) that the user wants to enter. To make useful predictions an accurate language model is necessary. There are a lot of different strategies that can be used to represent, train, and use the language model. An in depth review of different prediction techniques and their evaluation can be found in [2].

Language models are usually trained using large amounts of training data. This makes it possible to create a model that represents a particular language as much as possible. Such language models can be used to create a general purpose text prediction system. General purpose text predictor is expected to return reasonable predictions when any text is being typed. Presage is one example of such general purpose text predictor [3]. General purpose text predictors, however, might not be perfect for assistive communication technologies. First of all, it is hard to obtain large amounts of model training data. This becomes even more difficult if we want to create predictors for different languages. Another problem is that main source of training data is usually literature books. Literature books tend to contain more indirect speech. A language models trained on such data will therefore be somewhat biased towards indirect speech. People on the other hand mainly used direct speech during communication. Assistive communication technologies therefore should ideally use direct speech language models.

In this paper we present an alternative approach. The language model is trained using only a limited amount of sentences. Such model of course cannot fully represent the language but never the less has some advantages. The training data for such a model could be prepared for each patient individually. A relative or a social worker could type in only the sentences that would be most likely used by the patient. The training data could even be generated using modified sentences from questionnaires used to evaluate patients mental [4] or physical health. Moreover Huntington's disease usually causes some degree of mental degeneration [5] and limiting user word choices could actually help them express their ideas clearly. It has been shown in [6] that text predictors can be beneficial in other healthcare areas as well.

The biggest limitation of such language model is that the user might decide to enter words that are not present in the model training data. In such situations the text predictor will not produce any reasonable predictions. We propose solving this by using an additional semantic language model. Such model would contain the semantic relationships of the words. It has been shown in [7] that semantic information can improve speech recognition. Similar techniques can also be applied to improve text prediction.

Incorporating semantic information into a language model that could easily be used by text predictor is, however, very challenging. One way of incorporating semantic information into a language model is by assigning probabilities *P*(*w*_*i*_∣*w*) to all semantic relationships [8]. An alternative approach incorporates word meanings into an additional *n*-gram language model [9]. Both of these approaches however require a semantic database as well as a large training data set. However, obtaining a large training data set might be difficult. This is a big problem in our case because the main text predictor is trained using only a small limited data set. We avoid this problem by using the semantic information only as a source of synonyms, hypernyms, and hyponyms.

The remaining of the paper is structured as follows. The language models used by the text predictor and their evaluation setup are described in Section 2.  Section 3 describes the text predictor evaluation results.  Section 4 is the conclusion.

## 2. Materials and Methods

### 2.1. *n*-Gram Language Model

*n*-gram language models are one of the most popular ways to represent any given language. *n*-gram is a contiguous sequence of *n* words extracted from training data. During the training process the model is created by recording all unique *n*-grams found in the training data into a database. Each database entry stores the *n*-gram and its count, that is, the number of times that this particular *n*-gram has appeared in the training data set. Such model representation is very convenient for training because *n*-gram counts can be automatically collected from the training data.

When using *n*-gram model independence assumption is made so that each word depends only on the last *n* − 1 words. This means that prediction of a word *x*_*i*_ is based on *x*_*i*−(*n*−1)_,…, *x*_*i*−1_, where *n* is the cardinality of the model. In practice model with cardinality *n* also contains all models with lesser cardinality, that is, *n* − 1, …, 1. In this case each model returns a defined number of predictions and their probabilities. These probabilities are usually weighted to make sure that predictions from the model with higher cardinality are preferred. Each prediction returned by the model is a single word.

We used *n*-gram models to create the specialized text predictor for Huntington's patients. Medical health assessment questionnaires where used to prepare the training data. The training data was in English. These questionnaires contained questions about most often medical conditions and pain descriptions. Each question was manually converted into a statement because that is what the text predictor is expected to produce. This particular data set would be suitable for the use case where a doctor or relative is trying to evaluate patient's health condition. Additional data sets could easily be constructed for other use case scenarios.

**Figure 1:**
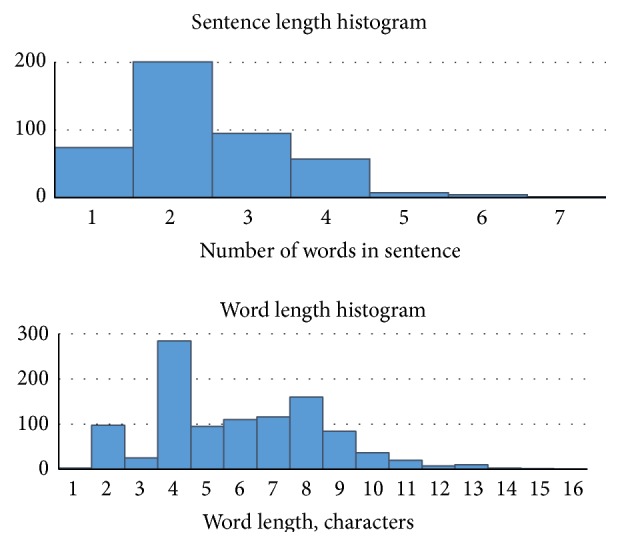
The statistics of the training data corpora: sentence length histogram and word length histogram.

The training data consisted of 439 statements (sentences) and 1056 words of which 420 are unique. The corpora statistics are provided in Figure 1. Longer statements usually describe full symptoms, for example, “reduced sensation in hands”. Short statements on the other hand mostly described feelings, for example, “weakness” or “intense pain”.

The model was evaluated using the same training data set because in our use case this is what we expect the user to be writing. For each word in the sentence we measured the percentage of characters (POC) that had to be provided for the text predictor before the predictor returned the correct word in the list of predictions. For each evaluated word the predictor was given up to *n* − 1 words preceding the predicted word. This was done to make sure that the predictor can use the model with cardinality *n* when calculating new word predictions.

The lowest possible POC for any given word is 0%. We get 0% POC when no letters of a currently predicted word have been entered and it is already shown in the text predictor predictions list. The highest possible POC on the other hand is 100%. In this case all of the current word letters had to be entered and current word was still not returned by the text predictor. Note that lower POC values are better.

After calculating the POC of each word in the test data set we calculated the total percent of characters (TPOC) for the text predictor using the weighted average formula. Each observation was weighted by the number of characters of that observation:

(1) $$\begin{array}{c} \text{TPOC}=\frac{\sum_{i=1}^{m}{POC}_{i}\times {nch}_{i}}{\sum_{i=1}^{m}{nch}_{i}}. \end{array}$$

Here nch_*i*_ is the number of characters of the *i*th word and *m* is number of words in the data set. We weighted the POC of each word according the number of characters of that word. This is done to show that POC of 50% for a 6-character word is a lot better than the POC of 50% for a 2-character word. In the former case the user could avoid typing 3 characters whereas in the latter case the user only avoids typing 1 character.

We also calculated the standard deviation (STD) of the TPOC measurements using the weighted standard deviation formula:

(2) $$\begin{array}{c} \text{STD}=\sqrt{\frac{\sum_{i=1}^{m}{nch}_{i}{\left({POC}_{i}-TPOC\right)}^{2}}{\left(\left(M-1\right)/M\right)\sum_{i=1}^{m}{nch}_{i}}}. \end{array}$$

Here *M* is the number of words used during the testing, namely, 1056. During the experiments we have also calculated confidence intervals (CI) at 95% confidence level.

### 2.2. Semantic Language Model

*n*-gram language models are based purely on word cooccurrence frequencies. These models are, however, not able to record the meaning of the modelled words. Semantic language models on the other hand try to encode the meaning of the language. Such models usually contain a database of binary relationships that join various language words. We use WordNet as a source of reliable semantic information [10].

As we discussed above, incorporating semantic information into the text predictor is a challenging task. We instead use sematic information to improve text input rate when the specialized *n*-gram text predictor fails to provide reasonable predictions. Our text predictor is trained using only a small set of specialized phrases that we expect the patient will be using. It is, however, likely that the user will type the phrases that have the same semantical meaning but will use different words. These situations cannot be handled using an *n*-gram text predictor, because these predictors can only use words provided during the training.

**Figure 2:**
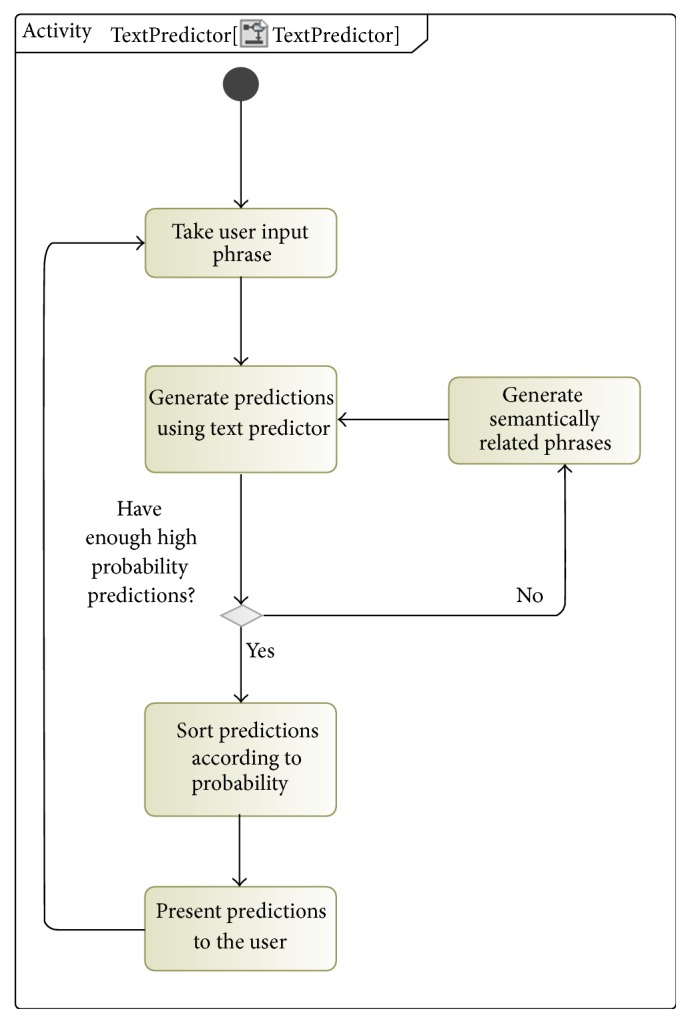
Semantic text predictor algorithm diagram.

For example, the *n*-gram predictor might have been trained to predict word “pain” after the word “hand” has been entered by the user. The user, however, might decide to enter word “arm” instead of “hand”. In this case the text predictor might not provide any reasonable predictions, because the phrase “arm pain” was not present in the training data set. We propose solving this problem by using semantic database to extract synonyms, hypernyms, and hyponyms of each word that the user has entered. This data can then be used to generate additional predictions from the specialized text predictor. Note that this approach would only be usable when the user has already entered a word that is not present in the text predictor training data set; that is, no predictions will be provided for completion of unknown words. The algorithm diagram is shown in Figure 2.

The algorithm works as follows:

1. Given the input phrase already entered by the user calculate the next predicted word using the specialized text predictor. Here each prediction is a word and its probability of being a desired completion.
2. Check if there are any predictions that have probabilities lower than a given threshold. If such predictions do not exist, the list of predictions is presented to the user. The thresholds were calculated automatically for each model cardinality. This was done by calculating the average probability of all the correct predictions.
3. Query the semantic database using the input phrase to extract semantically related words. For each word we extract its synonyms, hypernyms, and hyponyms. Here we discard extracted words that are not present in the unigram text predictor database, because these words will not produce any relevant predictions.
4. Use the remaining semantically related words to generate new predictions. All the predictions are then sorted according to their probability. The maximal allowed number of predictions is then returned to the user.

## 3. Results and Discussion

We used three different *n*-gram text predictors when performing the experiments. First, we used the “standard” *n*-gram language model that is provided with Presage [3]. This is a general purpose language model that is trained using literature books. Second, this standard model was “adapted” using our specialized training data set; that is, the model was further trained with our data set. The last model was a “specialized” language model that was trained only on the specialized data set.

Each model contains a data set of all unique *n*-grams that were extracted from the training data set. Training is performed by finding these *n*-grams and calculating the number of their occurrences. This data can then be used during the prediction step. Note that unigram model is only a database of all unique words and their occurrence counts. All other *n*-gram models generate all possible permutations of *n* words in each training sentence. For example, if we have a training sentence “constant numbness of legs” the following bigrams will be generated: “constant numbness” and “numbness of” and “of legs”. If a generated *n*-gram already exists in the model only its count is incremented. The training of “adapted” model is performed by adding new unique *n*-grams to the model and incrementing the counts for already existing *n*-grams.

During the experiments we calculate the POC for each word in the data set. When calculating the POC of the first word in each sentence the letters of that word are provided (one by one) until the predictor returns the word as one of predictions. For example, if the current word is “spine” and we had to provide “spi” before “spine” appears as one of the predictor outputs, then the POC of this word would be 60%. Up to *n* − 1 words (here *n* is model cardinality) are provided as a context for the predictor, but these words do not affect the POC of current word. POC can also be 0% for some words. There are two situations when this might happen. First, when the first word in a sentence is one of the most frequently found in training data and is always returned in a list of predictions. Second, when given the context a word is predicted without providing any letters of that word.

One example sentence from data set is “sudden weakness in limbs”. This sentence was queried using 3-gram model and 3 predictions. For the first word we need to provide “sud” and “sudden” was among the three predictions resulting in the POC of 50%. For second word we only had to provide “sudden” and “weakness” was predicted, that is, POC 0%. “in” also had POC of 0% after providing “sudden weakness”. Final word had a POC of 20%; that is, “weakness in l” was provided for predictor. The TPOC for this sentence would be 19.05%.

**Figure 3:**
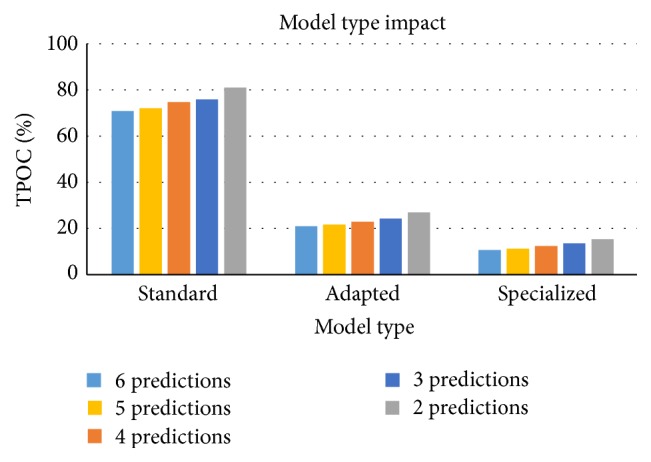
The TPOC of standard, adapted, and specialized text predictors. Each predictor was configured to return 6 to 2 predictions.

All three models where evaluated using the same specialized data set. Each text predictor was configured to calculate two to six predictions. Note that increasing the number of predictions will reduce the TPOC of the text predictor. However, it is preferable to have as little predictions as possible because it will make it easier for the user to select the desirable prediction. We believe that providing only one prediction or more than six predictions is impractical. The impact of the model type on the TPOC result is shown in Figure 3.

As we can see the “standard” language model had very high TPOC levels. Adapting the language model with the specialized training data set improved the text predictor performance significantly. The difference between the “adapted” model and the “specialized” model was less significant. Nevertheless, the user would have to enter almost two times fewer characters using the “specialized” model compared to the “adapted” model. Note that during these experiments 3-gram model cardinalities where used, because this was the cardinality of the “standard” language model. The results of this experiment are summarized in Table 1.

**Figure 4:**
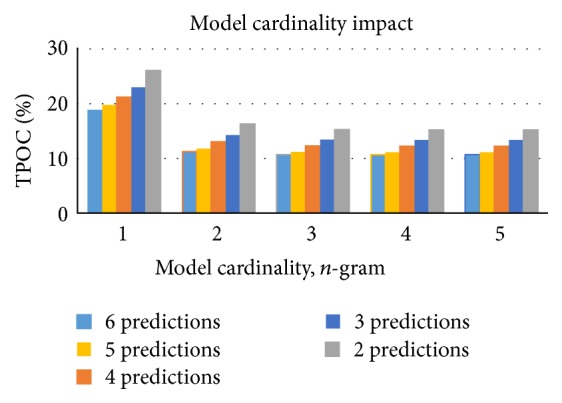
The TPOC of five language models with different cardinalities.

Another set of experiments was performed to study the impact of model cardinality. We trained five language models that had the cardinalities from 1 to 5. All models were trained and evaluated using the specialized data set. Note that, for example, a model of cardinality 3 internally also contains models with all possible lower cardinalities. The results of these experiments are provided in Figure 4.

As expected increasing model cardinality also improves its TPOC. The bigram model had a significantly improved TPOC compared to unigram model. The 3-gram model had a lot less significant improvement. Higher cardinality models had almost no noticeable TPOC improvement. Detailed results are provided in Table 2.

This experiment clearly shows that expanding model cardinality beyond 3 is not practical. It is of course worth noting that the majority of phrases used in the training data set contained 3 word phrases. We recommend choosing the cardinality of the model by examining the training data. Using cardinality higher than 3 is, however, rarely necessary.

**Figure 5:**
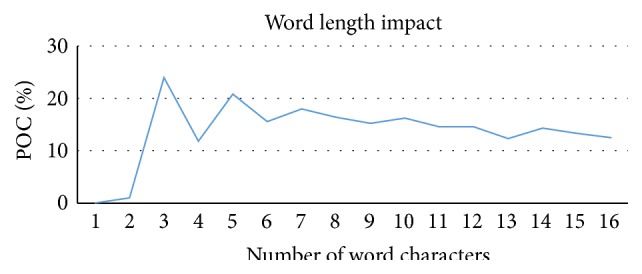
Word length impact on text predictor performance.

We also examined how word lengths influence the performance of the specialized text predictor. Specialized 3-gram text predictor was used during this experiment. The predictor was configured to only return two predictions. The test data set contained words that had variable lengths from 1 to 15 characters. Note that during this experiment we calculated POC instead of TPOC; that is, results are not weighted by word lengths. The experiment results are shown in Figure 5.

The experiment has demonstrated that text predictor has a sufficiently good performance even for different length words. This shows that the user will be able to type in long phrases efficiently. The POC curve jumps are most likely caused by the fact that we have varying amount of different length words present in training data set (see Figure 1).

## 4. Conclusions

This paper presented a design of a specialized text predictor for patients with Huntington's disease. We demonstrated that a specialized predictor with limited vocabulary can achieve significantly better performance than a standard general purpose text predictor. This is especially relevant for Huntington's patients, because they should be able to communicate their ideas quickly before losing focus.

One problem not considered in this paper is the order of the training data sentence words. If a system user decides to enter words in an order different from that in the training set the predictor might not generate good predictions. This problem could be addressed by automatically generating all possible permutations of all training sentences and using these for training the language model. It is, however, not yet clear how this would affect the predictor performance.

The text predictor was used to predict English language. The described techniques are generic and could be adapted to other languages as well. Models trained for languages with high inflection level would have lower performance. One alternative is to only predict word roots. This, however, would require nontrivial modifications in several algorithm steps.

This paper focused on creating a specialized text predictor for Huntington's patients. The predictor is intended to be used for diagnosing health condition. The described predictor could also be used by other patient's. They might, however, need a different training data set.

The study provides evidences supporting the usefulness of text prediction technology for Huntington's patients and justifying the importance of relevant research in terms of predicting algorithm and assistive user interface that are still lacking in the field. The proposed approach concentrated on Huntington's patients, but it could be easily applied in other domains as well.

## Figures and Tables

**Table 1:** Model type impact results.

Number of predictions		Model type		
		Standard	Adapted	Specialized
2	TPOC, %	81.05	26.9	15.31
	STD, %	25.61	24.93	16.98
	CI, %	1.54	1.5	1.02

3	TPOC, %	75.97	24.33	13.45
	STD, %	27.05	23.41	15.39
	CI, %	1.63	1.41	0.93

4	TPOC, %	74.81	22.93	12.29
	STD, %	28.05	22.72	14.41
	CI, %	1.69	1.37	0.87

5	TPOC, %	72	21.77	11.2
	STD, %	29.66	22.08	13.61
	CI, %	1.79	1.33	0.82

6	TPOC, %	70.89	20.85	10.72
	STD, %	30.31	21.54	13.25
	CI, %	1.83	1.3	0.8

**Table 2:** Model cardinality impact results.

Model cardinality		Number of predictions				
		2	3	4	5	6
1	TPOC, %	26.09	23.03	21.21	19.76	18.76
	STD, %	16.41	14.18	13.52	13.02	12.59
	CI, %	0.99	0.86	0.82	0.79	0.76

2	TPOC, %	16.31	14.28	13.01	11.8	11.23
	STD, %	16.7	15.23	14.31	13.55	13.22
	CI, %	1	0.92	0.86	0.82	0.8

3	TPOC, %	15.31	13.45	12.29	11.2	10.72
	STD, %	16.98	15.39	14.41	13.61	13.25
	CI, %	1.02	0.93	0.87	0.82	0.8

4	TPOC, %	15.21	13.39	12.24	11.15	10.7
	STD, %	17	15.39	14.41	13.61	13.25
	CI, %	1.03	0.93	0.87	0.82	0.8

5	TPOC, %	15.21	13.39	12.24	11.15	10.7
	STD, %	17	15.39	14.41	13.61	13.25
	CI, %	1.03	0.93	0.87	0.82	0.8
